# Atomoxetine for Orthostatic Hypotension in Traumatic and Non-traumatic Spinal Cord Injury: A Case Series From a Neuro-Rehabilitation Setting

**DOI:** 10.7759/cureus.116985

**Published:** 2026-09-26

**Authors:** Navin B Prakash, Shaneena Pavithran, Anupam Gupta, Meeka Khanna

**Affiliations:** 1 Department of Neurological Rehabilitation, National Institute of Mental Health and Neuro Sciences, Bangalore, IND

**Keywords:** atomoxetine, low blood pressure, myelopathy, orthostatic hypotension, spinal cord injury

## Abstract

Orthostatic hypotension (OH) following spinal cord injury (SCI) can significantly limit functional recovery by impairing participation in rehabilitation. Non-pharmacological techniques and pharmacological therapies, including fludrocortisone and midodrine, are conventionally used to manage OH. However, when these measures fail, atomoxetine has demonstrated benefit in other neurogenic OH populations. Evidence regarding the efficacy of this selective norepinephrine reuptake inhibitor is limited in neurogenic OH related to SCI. In this case series, we describe five patients with SCI who had symptomatic OH that did not respond to conventional pharmacological agents but showed clinical improvement with atomoxetine (dose range of 10 mg to 23 mg). Hence, atomoxetine can be considered a potential alternative therapeutic option for OH related to SCI in patients with OH refractory to conventional management.

## Introduction

Orthostatic hypotension (OH) is a common complication following spinal cord injury (SCI). It is characterized by a sustained decline in arterial blood pressure (BP) after transitioning to an upright position and is defined as a drop of >20 mmHg in systolic pressure or >10 mmHg in diastolic pressure within three minutes of standing or during tilt-table testing at ≥60° [1]. This reflects an inadequate autonomic and cardiovascular response to postural change, frequently encountered in cervical and high thoracic lesions [2]. The vagus nerve exits the brainstem and provides parasympathetic control to the heart; it remains intact in individuals with SCI. Descending sympathetic fibers provide sympathetic control of the heart and splanchnic vasculature. In high-level SCI (particularly above T6), these sympathetic pathways are disrupted, reducing sympathetic activity and impairing the pressor response to a fall in BP [3]. Additionally, other probable mechanisms that can lead to OH following a lesion in the spinal cord include altered control of the spinal sympathetic pathways, changes in peripheral baroreceptor sensitivity and control, loss of peripheral skeletal muscle pumping activity secondary to paralysis of lower limb muscles, cardiovascular dysfunction, especially in the acute period, and disturbances in salt and water balance [2,3]. Clinically, OH may present with dizziness, headache, visual disturbances, and syncope, thereby limiting mobilization, participation in therapy, and functional recovery [2].

Non-pharmacological strategies include medication review, patient education, adequate fluid and salt intake, compression stockings, abdominal binders, elevating the head of the bed by 10-20°, and gradual tilt-table training [3]. When these measures are ineffective, clinicians consider pharmacological therapy. Conventional first-line treatment includes fludrocortisone, a synthetic mineralocorticoid that increases plasma volume through sodium and water retention, and midodrine, an α-adrenergic agonist that increases peripheral vascular resistance [4]. However, these medications are of limited use due to adverse effects like supine hypertension, electrolyte imbalances, and inadequate symptom relief [4,5].

Atomoxetine is administered orally and is prescribed for the treatment of attention-deficit/hyperactivity disorder [6]. Its plasma half-life varies (5.2 to 21 hours) depending on individual metabolism (slow or fast) [6]. Due to its pressor effects, it has emerged as a potential alternative therapeutic option [7]. Jung et al. [8] demonstrated the efficacy of atomoxetine in managing primary OH in Parkinson’s disease and multiple system atrophy. It acts at peripheral postganglionic synapses to increase norepinephrine levels; these pathways remain intact in people with SCI [6]. Despite existing evidence supporting the role of atomoxetine in neurogenic OH, clinical data in patients with SCI remain limited.

The ASIA Impairment Scale (AIS) is used to classify SCI severity into complete (AIS A) or incomplete (AIS B to D), with categories C and D denoting a motor incomplete injury [9]. Individuals with AIS A have complete loss of sensory and motor function in the sacral segments (S2-S5) on clinical examination, as defined by absence of voluntary anal contraction, loss of deep anal pressure, and absence of sensations (light touch and pinprick). Sparing of sensory function in these sacral segments is categorized as AIS B [9]. The differentiation between AIS C and D is based on the relative preservation of muscle function below the neurological level of injury, wherein, in AIS C, less than 50% of the key muscles below the neurological level of injury have a muscle grade of ≥3, whereas in AIS D, at least 50% have a muscle grade of ≥3 [9].

This case series reports the use of atomoxetine in individuals with traumatic and non-traumatic SCI who did not respond to conventional agents.

## Case presentation

All the patients underwent detailed neurological assessment and baseline classification. BP was measured every four to six hours. Postural fluctuations in BP were formally assessed at baseline in all patients with myelopathy. Vital signs, including BP, were monitored during physiotherapy and occupational therapy sessions. When patients showed signs or symptoms of autonomic disturbance, BP was recorded supine and at one-minute and three-minute intervals after transitioning to a sitting position. The treating clinician determined the initial dose of atomoxetine (5 mg or 10 mg in the morning) and titrated it based on clinical response, as described below.

Case 1

A 43-year-old man with no known comorbidities presented with tetraplegia of two months' duration secondary to a traumatic cervical SCI following a road traffic accident. On assessment, his neurological status was classified as C4 AIS C, with generalized hypotonia in both upper limbs (ULs) and lower limbs (LLs). Motor examination showed Medical Research Council (MRC) grade 2/5 power in the proximal muscle groups and 1/5 power in the distal muscle groups of both ULs and 0/5 power in both LLs. He reported dizziness, lightheadedness, and blurred vision when sitting upright from a supine position. On examination, an orthostatic drop in BP was noted. Bilateral compression stockings were applied up to mid-thigh, and gradual tilt-table training was initiated. Because of a persistent, significant drop in BP, he was started on oral fludrocortisone (100 mcg daily in divided morning and afternoon doses). On the fourth day of pharmacotherapy, the patient exhibited consistently elevated BP recordings (around 160/123 mmHg). These BP readings were not accompanied by bradycardia, diaphoresis, or identifiable noxious stimuli, effectively ruling out autonomic dysreflexia and raising concerns of treatment-induced supine hypertension; hence, fludrocortisone was discontinued. He was initiated on oral atomoxetine (18 mg in the morning) along with tapering of midodrine. Oral fluid intake was monitored, and other non-pharmacological measures were continued. After 10 days, an additional 5-mg post-lunch dose of atomoxetine was added, increasing the total daily dose to 23 mg; symptoms improved markedly, and tolerance to an upright posture improved. The patient could sit for about 30 minutes and stand for five minutes. However, due to the need for increased oral fluids, bladder management continued with an indwelling urethral catheter.

Case 2

An 18-year-old man with no known comorbidities presented with a three-month history of post-infectious Epstein-Barr virus long-segment myelitis extending from the cervicomedullary junction to the T8 level. At admission, he had hypotonia in the bilateral LLs, MRC grade 4/5 power in the right UL, 3/5 power in the left UL, and 0/5 power in the bilateral LLs. His neurological status was classified as C5 AIS B. He reported dizziness and a loss of consciousness lasting a few seconds when transitioning from a supine to a sitting position, and physical examination confirmed OH. Because the symptoms did not resolve with non-pharmacological measures, he was started on oral fludrocortisone and midodrine. However, due to persistent OH, these medications were tapered and discontinued. He was subsequently started on oral atomoxetine (5 mg in the morning). After three days, the dose was increased to 10 mg, after which the OH subsided. However, the patient had recurrence of symptoms three weeks later during therapy; hence, an afternoon dose of 5 mg was added (total daily dose of 15 mg). Thereafter, he sat upright without symptoms for one hour and actively participated in therapeutic standing with bilateral lower limb orthoses.

Case 3

A 52-year-old man with no known comorbidities presented for inpatient rehabilitation 10 days after undergoing surgery for C3-C6 ossification of the posterior longitudinal ligament. His neurological status was C3 AIS C tetraplegia. Examination revealed hypertonia in both LLs, with MRC grade 1/5 power in the right UL and LL and 2/5 power in the left UL and LL. He reported episodes of dizziness lasting a few seconds when rising from supine to sitting. Non-pharmacological strategies did not resolve his OH; hence, oral fludrocortisone was initiated. However, the medication was gradually tapered and discontinued in view of treatment-induced hypokalemia. He was subsequently started on oral atomoxetine (10 mg single morning dose). Three days after initiating atomoxetine, the OH subsided, with no associated electrolyte imbalances. At discharge, he was independent in feeding, grooming, and brushing with a universal cuff.

Case 4

A 55-year-old man with C4 AIS C tetraplegia secondary to a traumatic C3-C4 prolapsed intervertebral disc presented for inpatient rehabilitation two months postoperatively. Neurological examination showed hypertonia in all four limbs, MRC grade 3/5 power in the proximal group and 2/5 power in the distal group of muscles of the bilateral ULs, and 1/5 power in the right LL and 2/5 power in the left LL. He was noted to have asymptomatic OH. He was prescribed oral fludrocortisone; however, he developed supine hypertension within three days of therapy, and hence it was tapered and discontinued. After one week of treatment with an abdominal binder and oral atomoxetine (10 mg), the OH subsided, with no evidence of medication-induced hypertension on monitoring. At discharge, he could feed and brush independently using an orthosis.

Case 5

A 49-year-old man with comorbid hypothyroidism presented for rehabilitation two months following surgical intervention for a traumatic C5-C6 prolapsed intervertebral disc. His neurological status was classified as C7 AIS C tetraplegia. Neurological examination revealed hypertonia in both LLs, MRC grade 5/5 power in the proximal muscle groups and 3/5 power in the distal muscle groups of the bilateral ULs, and 2/5 power in the bilateral LLs. He presented with OH accompanied by episodes of syncope and blurring of vision when transitioning to an upright sitting position and during tilt-table training above 40 degrees. He was managed with oral fludrocortisone and midodrine; however, due to severe hypokalemia (2.7 mmol/L) and financial constraints, these medications were discontinued. Oral atomoxetine (10 mg) was prescribed. After two days, his OH improved, with no associated side effects.

All patients were asymptomatic at the three-month post-discharge follow-up. Table 1 summarizes the clinical characteristics, pharmacotherapeutic management, functional outcomes (SCIM-III scores), and longitudinal follow-up for all five cases.

**Table 1:** Clinical characteristics and management of patients T: traumatic, NT: non-traumatic, NL: neurological level of injury, AIS: ASIA Impairment Scale, DOI: duration of injury, BP: blood pressure (expressed in mmHg), SCIM-III: Spinal Cord Independence Measure-III

Case no.	Etiology	NL and AIS grade	DOI (months)	Symptoms	Supine BP (mmHg)	Sitting BP at 1 min (mmHg)	Sitting BP at 3 min (mmHg)	SCIM-III admission	Atomoxetine total daily dose (mg)	Atomoxetine dosing (mg) schedule	Post-treatment supine BP (mmHg)	Post-treatment sitting BP at 3 min (mmHg)	SCIM-III discharge	Follow-up (months)
1	T	C4, AIS C	2	Dizziness, lightheadedness and blurring of vision	101/60	77/42	74/36	17	23	18 (morning), 5 (afternoon)	94/68	92/64	23	6
2	NT	C5, AIS B	3	Dizziness and loss of consciousness	88/54	76/42	66/32	20	15	10 (morning), 5 (afternoon)	100/60	94/58	31	3
3	NT	C3, AIS C	6	Dizziness	102/74	88/69	80/64	17	10	10 (morning)	115/70	110/67	23	3
4	T	C4, AIS C	5	Asymptomatic	130/87	100/60	70/40	11	10	10 (morning)	122/74	104/67	37	3
5	T	C7, AIS C	2	Syncope and blurring of vision	94/61	70/46	72/50	11	10	10 (morning)	114/80	102/70	46	3

## Discussion

OH is a common complication after SCI; loss of thoracic sympathetic outflow impairs vasoconstriction, causing venous pooling and reduced systemic vascular resistance when a person stands upright [1-3]. Effective management of OH is necessary to aid in early mobilization and facilitate participation in rehabilitation [4]. Common pharmacological agents include fludrocortisone and midodrine. However, their use could be limited due to side effects like supine hypertension, hypokalemia, and fluid overload [4,5].

This case series includes patients with cervical SCI with incomplete myelopathy (AIS B and AIS C). Wang et al. [10] demonstrated variable orthostatic cardiovascular responses in SCI patients, suggesting that neurological classification or SCI completeness cannot predict neurogenic OH alone. This reflects a distinct autonomic-somatic dissociation, in which descending sympathetic pathway inputs are disrupted despite partial preservation of adjacent motor pathways, resulting in BP instability across various AIS categories. In this case series, presentation varied across patients, although all had incomplete injury. This highlights the need for an individualized assessment and treatment of neurogenic OH.

Furthermore, the baseline frequency of symptomatic OH is influenced by the underlying etiology and mechanism of SCI [11]. Compared with compressive myelopathy (degenerative or post-infectious), the risk of developing severe OH is greater in traumatic SCI [12]. In non-traumatic SCI, the neurovascular system activates certain compensatory mechanisms, such as the gradual upregulation of peripheral vascular receptors and localized spinal-level postural reflexes. These mechanisms may protect patients from sudden hemodynamic changes as seen in acute SCI.

When conventional pharmacotherapy fails or leads to intolerable side effects, targeting the norepinephrine transporter (NET) with atomoxetine may provide a site-specific response. A selective norepinephrine reuptake inhibitor such as atomoxetine can increase synaptic norepinephrine and improve sympathetic tone [6,7]. Selective NET inhibition depends on the anatomical and functional integrity of the postganglionic sympathetic apparatus [6,13]. Because SCI and non-traumatic myelopathies represent purely preganglionic disruptions of central autonomic pathways, peripheral postganglionic noradrenergic fibers remain intact and can synthesize local neurotransmitters [6,7]. By blocking NET at these peripheral junctions, atomoxetine prevents norepinephrine reuptake, maximizing its availability at the neuro-effector junction and restoring vascular tone and sympathetic pressor responses [6,7,13].

This precise anatomical site of sympathetic disruption explains why atomoxetine yields divergent outcomes in neurogenic OH. Ramirez et al. [14] reported that atomoxetine may produce a greater pressor response than midodrine in the management of OH with severe autonomic failure, which included cases of pure autonomic failure, multiple system atrophy, and Parkinson’s disease. However, in disorders with preserved peripheral postganglionic fibers, such as multiple system atrophy, the pressor response produced by atomoxetine may match or exceed that of direct alpha-1 agonists [8,15]. Conversely, in primary degenerative alpha-synucleinopathies with postganglionic noradrenergic denervation, such as advanced Parkinson’s disease or pure autonomic failure, atomoxetine monotherapy often fails due to the loss of the cellular targets on which it normally acts [7,8]. In these peripherally denervated states, pairing atomoxetine with a ganglionic enhancer like pyridostigmine may elicit a synergistic pressor response [16]. This limitation does not affect the present cases with preganglionic lesions (SCI or non-traumatic myelopathy).

All patients included in this case series received fludrocortisone initially; however, it was tapered and stopped in view of lack of response, supine hypertension, or hypokalemia. In some cases, midodrine was not feasible due to financial constraints. In our cases, atomoxetine improved OH without significant side effects (costing about $1 for 10 tablets). Atomoxetine doses ranged from 10 mg to 23 mg. This dose range may partly reflect variability in drug metabolism among individuals (slow or fast), leading to higher blood levels in people with slower metabolism [6]. We did not observe an immediate pressor response to atomoxetine (i.e., within a few hours) [6]. Rather, our patients responded after approximately 48-72 hours; this may reflect the need for sufficient plasma and synaptic concentrations to induce the desired pressor response in patients with SCI who develop OH through multiple pathophysiological mechanisms.

In a recent trial, data from a heterogeneous neurogenic population suggested that chronic four-week atomoxetine monotherapy may lead to receptor downregulation or counter-regulatory mechanisms that limit its long-term sympathetic response compared with placebo [17]. However, in the present cases, we did not observe a reduced pressor response with atomoxetine monotherapy; this may reflect preserved postganglionic pathways in our patients. In a previous report, customized low-dose atomoxetine administration maintained BP over 10 weeks in a patient with OH refractory to conventional mineralocorticoids and direct alpha-analogs [18].

Ultimately, targeted management of OH in neuro-rehabilitation must move away from broad diagnostic labels or motor indices. By recognizing the etiological nuances of myelopathy, atomoxetine can be considered an alternative therapeutic option in patients with SCI who do not respond adequately to conventional agents.

Certain limitations warrant consideration. Tilt-table BP measurements were performed but not recorded consistently; therefore, they were omitted. Second, we did not perform continuous ambulatory BP monitoring. Finally, patients' intake of caffeinated beverages and other dietary stimulants that could affect autonomic function was not controlled.

## Conclusions

Managing OH after SCI is important to support rehabilitation progress. This case series suggests that atomoxetine may be a well-tolerated and effective therapeutic agent for the management of OH in individuals with traumatic and non-traumatic SCI. Atomoxetine may be particularly helpful for those who experience side effects or have an inadequate response to non-pharmacological measures and conventional pharmacotherapy. Future prospective studies are essential to determine the impact of atomoxetine on OH in the SCI population.
